# Professional Attitudes and Practice of Pediatric Dentists About the Use of Local Anesthesia for the Treatment of Children Under General Anesthesia

**DOI:** 10.5812/aapm-143076

**Published:** 2024-03-17

**Authors:** Reyhaneh Faghihian, Aryana Golabbakhsh, Elahe Asnaashari

**Affiliations:** 1 Department of Pediatrics, Dental Research Center, Dental Research Institute, School of Dentistry, Isfahan University of Medical Sciences, Isfahan, Iran; 2 Dental Students Research Committee, School of Dentistry, Isfahan University of Medical Sciences, Isfahan, Iran; 3 Department of Pediatrics, School of Dentistry, Isfahan University of Medical Sciences, Isfahan, Iran

**Keywords:** Attitude, General Anesthesia, Local Anesthesia, Pediatric Dentistry, Professional Practice

## Abstract

**Background:**

The application of local anesthesia in dental surgeries conducted under general anesthesia poses a challenge in pediatric dentistry. There is a lack of consensus regarding the benefits and drawbacks of using general anesthesia in this field.

**Objectives:**

The purpose of this study was to assess the attitudes and practices of pediatric dentists regarding the use of local anesthesia for dental treatments in children under general anesthesia in Iran.

**Methods:**

This cross-sectional study involved 110 pediatric dentists from across Iran. The dentists' professional attitudes and practices were assessed using a specially designed questionnaire for this study. The questionnaire was distributed through the Line press system, and the data were analyzed following collection.

**Results:**

Regarding professional practices, 34.5% of specialists refrained from using local anesthesia. The most frequent application of local anesthesia was observed in tooth extractions. The preferred local anesthetic agent was 2% lidocaine with 1:100 000 epinephrine. A lower dose than that used in outpatient settings was administered, and most specialists allowed sufficient time for the anesthesia to take effect.

**Conclusions:**

This study revealed that opinions and attitudes towards the use of local anesthesia in the dental treatment of children under general anesthesia vary across different specialties and are significantly influenced by the patient's condition, type of treatment, and practice techniques.

## 1. Background

Pain management is now a significant factor in reducing anxiety compared to earlier times. One challenge encountered by families and pediatric dentists is children's fear of dental procedures (1). This fear and anxiety can lead to adverse outcomes for patients, including avoidance of regular dental visits, which may result in issues like pain, abscesses, and the loss of both primary and permanent teeth (2). Research on addressing fear and anxiety in treating anxious children has explored the use of tranquilizers, narcotics, behavior control techniques, or a combination thereof (3-5). However, behavior control techniques come with limitations, notably their potential ineffectiveness at higher levels of anxiety (6). Combining pharmaceutical management with behavior control methods in pediatric dentistry enhances the success rate and quality of treatments. Nevertheless, drugs that reduce respiratory rate and inhibit the gag reflex carry certain risks (7). Therefore, it is advised that this approach be reserved for cooperative children with minimal dental treatment needs and patients who have contraindications for general anesthesia (8).

Despite recent advancements in medications and techniques for general anesthesia, postoperative agitation remains a significant complication that necessitates careful attention to its diagnosis, treatment, and reduction in the recovery room (9, 10). This issue, along with others, underscores the importance of addressing the adverse effects of general anesthesia. Utilizing local anesthesia is one effective strategy for minimizing the negative complications associated with general anesthesia (11). Typically, local anesthesia reduces bleeding at the surgical site, eases the handling of soft tissues, and diminishes pain and systemic effects in comparison to other analgesics (12, 13). Critics of this technique argue that local anesthetic agents can increase the risk of lip biting, prolong the duration of the procedure, and raise the rates of toxic and allergic reactions, as well as alter heart rate during the procedure (13). Discrepancies exist among dental practitioners regarding their professional attitudes and practices about the use or non-use of local anesthesia during pediatric dental procedures under general anesthesia. Moreover, some practitioners are more inclined to use local anesthesia for various reasons. 

## 2. Objectives

Given the scarcity of comprehensive studies on this topic in our country, the current study was conducted to thoroughly investigate the factors influencing the use of local anesthesia during general anesthesia for pediatric dental procedures, employing a questionnaire to assess the attitudes and practices of pediatric dentists.

## 3. Methods

This descriptive, cross-sectional study was conducted in 2020 with 110 pedodontists across Iran. Participants were provided with a written explanation of the study conditions, and the confidentiality of their data was guaranteed. The study's protocol received approval from the Ethics Committee of Isfahan University of Medical Sciences under the code IR.MUI.RESEARCH.REC.1399.791. Participants were randomly chosen from dentists nationwide through a simple convenient sampling method until the required sample size of n = 110 was reached. This size was determined based on a 95% confidence interval (Z_1-a/2_ = 1.96), a study power of 80% (Z_1-b_ = 0.84), a standard deviation of approximately 1.7 (14) regarding attitudes towards the use of local anesthesia during general anesthesia for pediatric dental procedures, and an error rate of 0.45.


$$N=\frac{{\left(Z_{1-\frac{a}{2}}+Z_{1-b}\right)}^{2}*{\left(S\right)}^{2}}{D^{2}}=\frac{{\left(1.96+0.84\right)}^{2}*{\left(1.7\right)}^{2}}{{\left(0.45\right)}^{2}}=110$$

A questionnaire developed by the researchers was employed to conduct the study. The questionnaire's items were inspired by a similar article (15) and modified after review in a meeting with one anesthesiologist, three pedodontists, and one community dentist. Preliminary tests were conducted with a group equal to at least 10% of the sample size, chosen randomly, to assess the questionnaire's reliability. These tests were analyzed using SPSS software version 26, resulting in a Cronbach's alpha reliability coefficient of 0.81 for the questionnaire. To ensure the questionnaire's validity, the draft was reviewed by several members of the pediatric dentistry department, and their feedback was incorporated into the final version.

All pedodontists across the nation who performed pediatric dental procedures under general anesthesia and were willing to participate were eligible for inclusion in this study. Those who did not perform pediatric dental procedures under general anesthesia were excluded. The researcher-developed questionnaire was distributed to participants online.

Once the questionnaire was finalized, a comprehensive list of pedodontists nationwide was compiled. From this list, 110 pedodontists were randomly chosen, and they received the questionnaire along with a message encouraging them to complete it and relevant instructions through the Pors online system. During the questionnaire collection process, any specialist who failed to complete the questionnaire despite receiving a reminder was excluded. A replacement was then selected to ensure a total of 110 completed questionnaires. Incomplete responses led to the exclusion of the questionnaire, and the respective participant was replaced by another.

The questionnaire was divided into three sections. The first section collected personal information, including age, work experience, and the location of practice. The second section contained 15 questions regarding attitudes towards the use of local anesthesia during dental procedures under general anesthesia, organized into four domains: Effects during the procedure (4 questions), effects after the procedure (5 questions), procedural steps (3 questions), and safety concerns (2 questions). Responses in the attitude section were recorded on a Likert scale with options ranging from “I strongly agree” to “I strongly disagree.” The third section focused on the professional practices of pedodontists in relation to local anesthesia, including 4 multiple-choice questions about the use of local anesthesia and the types of medication employed to descriptively evaluate the dentists' professional performance.

The content of the questionnaire was adapted from a similar study and refined in a session attended by one anesthesiologist, three pedodontists, and one specialist in community dentistry. A pilot study involving at least 10% of the sample size was conducted randomly to establish the questionnaire's validity, and the results were analyzed using SPSS software. The Cronbach’s alpha reliability coefficient for the questionnaire was determined to be 0.81. To further ensure reliability, the draft was reviewed by several faculty members in the Department of Pediatric Dentistry, incorporating their feedback to achieve a reliability score of 0.79.

As the questionnaire did not specify a cut-off point for the use or non-use of local anesthesia during general anesthesia but rather assessed pedodontists' attitudes, the data were presented descriptively, showing frequencies and percentages. The chi-squared test and Fisher’s exact test were employed to compare the frequency of local anesthesia use based on demographic data, with a 95% confidence interval. Data analysis was performed using SPSS 26 (IBM Corp., USA).

## 4. Results

Among the participants, 57 (51.8%) were male, while the remainder were female. In terms of age, 56 (59.1%) were between 25 and 35 years old, 40 (36.4%) were between 36 and 46 years old, and 5 (4.5%) were older than 45 years. Table 1 displays the frequencies of other basic data, including work experience and practice location.

**Table 1.** The Distribution of the Demographic Data of the Pedodontists Evaluated

Variables	Frequency (%)
**Work experience (y)**	
< 5	45 (4.9)
5 - 9	30 (27.3)
10 - 19	23 (20.9)
≥ 20	12 (10.9)
**Practice location**	
Public sector	36 (33.7)
Private office	20 (18.2)
Both	54 (49.1)

Table 2 shows the frequency of local anesthesia use according to demographic data. The results indicated no significant correlation between the use of local anesthesia and the various variables (P > 0.05).

**Table 2.** The Frequency Distribution of Using Local Anesthesia in Terms of Demographic Characteristics

Variables	Use of Local Anesthesia Administration, No. (%)		Sig. ^a^
	Yes	No	
**Age (y)**			0.370
25 - 35	20 (30.8)	45 (69.2)	
36 - 45	17 (42.5)	23 (57.5)	
> 45	1 (20)	4 (80)	
**Sex**			0.497
Male	18 (31.6)	39 (68.4)	
Female	20 (37.7)	33 (62.3)	
**Work experience (y)**			0.110
< 5	16 (35.6)	29 (64.4)	
5 - 9	6 (20)	24 (80)	
10 - 19	12 (52.2)	11 (47.8)	
≥ 20	4 (33.3)	8 (66.7)	
**Practice location**			0.450
Public sector	13 (36.1)	23 (63.9)	
Private office	9 (45)	11 (45)	
Both	16 (29.6)	38 (70.4)	
Others	8 (36.4)	14 (63.6)	

^a^ Statistical significance level was calculated using the chi-squared and Fisher’s tests at a 95% confidence interval.

Table 3 details pedodontists' attitudes toward the use of local anesthesia during general anesthesia. The table reveals that the majority of pedodontists did not believe that local anesthesia prolonged the duration of general anesthesia, increased patients' pain perception, or disrupted their vital signs. However, they did agree that local anesthesia reduced bleeding. Most pedodontists were of the opinion that local anesthesia lessened pain and shortened recovery time. They also thought that local anesthesia contributed to improved treatment quality, increased lip biting, and agitation. About 43% of pedodontists were against using bilateral inferior alveolar nerve blocks during general anesthesia, preferring unilateral IANB instead. Furthermore, most participants believed that the use of local anesthesia would reduce the likelihood of complications from general anesthetic agents and the dosage of local anesthesia. They also considered the risk of patient overdose with local anesthetic agents during general anesthesia to be higher than when such agents are used in an outpatient setting.

**Table 3.** The Frequencies of Pedodontists’ Attitudes Toward the Use of Local Anesthesia During General Anesthesia

Items	Responses, No. (%)		
	Agreement	Neutral	Disagreement
**The effects of the use of local anesthesia**			
Increased vital sign disturbance	41 (37.2)	21 (19.1)	48 (43.7)
Better bleeding control	61 (55.5)	19 (17.2)	30 (27.3)
Increased pain perception	39 (35.5)	16 (14.5)	55 (50.0)
Prolongation of general anesthesia	28 (25.5)	28 (25.5)	54 (49.0)
**The effects of administering local anesthesia**			
Decreased pain	71 (64.6)	15 (13.6)	24 (21.8)
Increased treatment quality	29 (26.4)	26 (23.6)	55 (50.0)
Shorter recovery	32 (29.1)	36 (32.7)	42 (38.2)
Increased lip biting	51 (46.3)	28 (25.5)	31 (28.2)
Increased agitation	29 (26.4)	31 (28.1)	50 (45.5)
**Administration of local anesthesia**			
Only extraction	33 (30.0)	29 (26.4)	48 (43.6)
Bilateral inferior alveolar block	48 (43.6)	31 (28.2)	31 (28.2)
Unilateral inferior alveolar block	42 (38.1)	30 (27.3)	38 (34.6)
**Patient safety during local anesthesia**			
Decrease the volume of LA when used in GA	47 (42.7)	24 (21.8)	39 (35.5)
Decreased odds of complications	43 (39.1)	37 (33.6)	30 (27.3)
Increased risk of overdose	56 (50.9)	28 (25.5)	26 (23.6)

Table 4 shows the practices of pedodontists regarding the use of local anesthesia during general anesthesia. According to the table, 34.5% of the pedodontists assessed did not utilize local anesthesia during general anesthesia.

**Table 4.** The Frequencies of Pedodontists’ Practical Approaches Toward the Use of Local Anesthesia During General Anesthesia

Questions and Choices	Frequency (%)
**Administration of local anesthesia during general anesthesia**	
I never administer it	38 (34.5)
For tooth extraction	22 (20)
For composite resin restorations of anterior teeth	20 (18.2)
For all procedures	19 (17.3)
Only for procedures that traumatize soft tissues	11 (10)
**Dentists waiting for the effect of local anesthetic agents to begin the procedure**	
I never administer it	38 (34.5)
Never	12 (10.9)
Seldom	7 (6.4)
Sometimes	15 (13.6)
Mostly	27 (24.5)
Always	11 (10)
**Using a lower dose of local anesthetic agents than in outpatient settings**	
I never administer it	38 (34.5)
Never	3 (2.7)
Seldom	9 (8.2)
Sometimes	14 (12.7)
Mostly	33 (30)
Always	12 (10.9)
**The type of the local anesthetic agent, if indicated**	
Local anesthetic agent with epinephrine	46 (41.8)
Local anesthetic agent without epinephrine	25 (22.7)
Long-acting local anesthetic agent with epinephrine	23 (20.9)
Long-acting local anesthetic agent without epinephrine	16 (14.5)

## 5. Discussion

The application of local anesthesia in children undergoing dental treatments under general anesthesia remains a contentious issue within pediatric dentistry. Some researchers and specialists in this area, as well as in related fields such as anesthesiology, argue that local anesthesia can reduce postoperative complications, including pain and the duration of recovery room stays, making it beneficial for patients; thus, they advocate for its use during dental treatment under general anesthesia. Conversely, other specialists caution that the combined dosage of local and general anesthetic agents in the patient’s system might heighten the risk of hemodynamic disturbances intraoperatively. As a result, they advise against the use of local anesthetic agents during general anesthesia. Meanwhile, numerous studies have explored the pros and cons of administering local anesthesia prior to general anesthesia, yielding mixed outcomes.

In this study, approximately 65.5% of pedodontists utilized local anesthesia, possibly due to their belief in its benefits, such as reducing intraoperative bleeding and postoperative pain upon awakening (noted by the highest percentage of respondents), enhancing the quality of dental procedures, and reducing the dosage and duration of general anesthesia required. Conversely, 34.5% of the participants refrained from using local anesthesia, citing concerns over the potential for prolonged general anesthesia, trauma to soft tissues (such as cheek and tongue biting and bleeding), disturbances in vital signs, listlessness in children after awakening, and an increased risk of overdose compared to outpatient treatments. The study highlighted varying attitudes among dental practitioners towards the use of local anesthesia during dental procedures under general anesthesia across different domains. Regarding intraoperative effects, the greatest consensus was on the efficacy of local anesthetics in controlling bleeding, while the most significant disagreement concerned their effectiveness in managing pain perception.

In the postoperative domain, the strongest agreement was on the reduction of pain after recovery, and the most disagreement was on the increased incidence of children's listlessness post-awakening. Concerning procedural aspects, the consensus was highest against the use of bilateral inferior alveolar nerve blocks, and the greatest disagreement was with the limitation of local anesthesia to tooth extractions during general anesthesia. Lastly, in the safety aspect of the procedure, the most agreement was on the heightened risk of local anesthetic agent overdose during general anesthesia compared to outpatient settings.

In a study conducted by Townsend et al. in Florida, the majority of participants utilized local anesthesia during general anesthesia in more than 90% of cases, citing its benefits in stabilizing vital signs and reducing the depth of general anesthesia during procedures (16). Das et al. explored the knowledge, attitudes, and professional practices of pedodontists regarding the use of local anesthesia. The findings revealed that 76.2% of dentists harbored negative attitudes towards the use of local anesthetic agents, perceiving them as hazardous for procedures conducted under general anesthesia, with 84% indicating that they did not employ local anesthesia (17). Regrettably, most participants in that study possessed limited knowledge about the benefits and drawbacks of local anesthesia. A comparison between the results of the aforementioned study and the current study indicates a more favorable perspective and practice concerning the use of local anesthesia in the latter.

According to the current study, nearly 60% of participants believe that administering local anesthesia prior to general anesthesia could reduce bleeding. Supporting this view, McWilliams and Rutherford demonstrated that the preoperative use of local anesthetic agents lessened postoperative hemorrhage, aligning with the opinions of specialists in the current study. However, they posited that it did not mitigate postoperative pain, contrasting with the perspectives of pedodontists in this study (18). Atan et al. assessed the impact of local anesthetic agents used during general anesthesia, concluding that local anesthesia could serve as an effective method to minimize complications associated with dental procedures, including pain (19).

The findings of the current study indicate that approximately 45% of pedodontists believe that local anesthesia reduces postoperative agitation, while 40% disagree with this view. In line with this, Jurgens et al. reported that the application of local anesthetic agents following general anesthesia led to increased calmness in children and reduced pain (20), aligning with the perspectives of many dentists in this study.

The results also reveal that nearly 65% of pedodontists are of the opinion that administering local anesthesia prior to procedures under general anesthesia reduces postoperative pain. In research conducted by De Verbizier et al. in France, local anesthesia was used during general anesthesia in more than half of the oral surgeries, leading to improved pain management and reduced preoperative and postoperative bleeding. For the remainder of the patients, local anesthesia was not used due to concerns about toxic and allergic reactions (15). Contrarily, Coulthard et al. (as cited by Townsend et al.) found that local anesthetic did not influence postoperative pain (21), differing from the views held by pedodontists in the current study. Such variations in findings might be attributed to differences in the training and experience of individuals regarding clinical study outcomes.

In a randomized, prospective study, Townsend et al. (as cited by Batarseh) concluded that administering local anesthesia after general anesthesia does not lessen pain or shorten the recovery duration; instead, it may lead to negative outcomes, such as an increased incidence of lip and cheek biting (13). According to these results, most participants did not believe that local anesthesia hastened patients’ recovery, with only about 29% acknowledging its effectiveness in reducing recovery time. Furthermore, a limited number of dentists believed in its role in minimizing lip biting (approximately 46%). However, only a few dentists in Townsend et al.’s study cited the aforementioned issues as reasons for not using local anesthesia, with other factors including the necessity for systemic analgesics to manage pain (21).

Other responses indicated that dentists' attitudes towards the use of local anesthesia were neutral, suggesting that many dentists believe its application should be based on the patient's condition and the specifics of the procedure. It's important to note that this study was not designed to definitively support or oppose the use of local anesthesia during procedures performed under general anesthesia. The variables and factors evaluated in the questionnaire have been discussed in previous studies, which have shown varying results. Hence, it can be inferred that a larger proportion of pedodontists in this study recognized the advantages of local anesthesia.

When examining the practical approaches of pedodontists towards the use of local anesthesia in conjunction with general anesthesia, it was found that 34.5% of them never utilized local anesthesia. Additionally, the most common application of local anesthesia was for tooth extractions. Nearly half of the pedodontists opted for lower doses of anesthetic agents during general anesthesia compared to what is typically used in outpatient settings, either often or always, with local anesthetic containing epinephrine being the most commonly chosen solution.

### 5.1. Conclusions

This study highlighted that attitudes towards the application of local anesthesia during pediatric dental procedures under general anesthesia varied across several domains, primarily influenced by the patient’s condition, the type of procedure, and the injection technique employed. The findings underscore the necessity for standardized guidelines regarding the use of local anesthetics in dental treatments performed under general anesthesia.

## Data Availability

The dataset presented in the study is available on request from the corresponding author during submission or after publication.
